# Endocytic Trafficking of DMP1 and GRP78 Complex Facilitates Osteogenic Differentiation of Human Periodontal Ligament Stem Cells

**DOI:** 10.3389/fphys.2019.01175

**Published:** 2019-09-12

**Authors:** Annette Merkel, Yinghua Chen, Anne George

**Affiliations:** Department of Oral Biology, The University of Illinois at Chicago, Chicago, IL, United States

**Keywords:** stem cells, molecular biology, cell biology, protein expression, matrix biology, mineralized tissue/development

## Abstract

Periodontal ligament contains periodontal ligament stem cells that maintain tissue homeostasis. Targeting hPDLSCs (human periodontal ligament cells) is a promising strategy for repair and regeneration of bone tissue destroyed by periodontal diseases. However, the mechanisms by which PDLSCs differentiate into osteoblasts to form a mineralized matrix is unclear. In this study, we demonstrate for the first time the molecular events that contribute to osteogenic differentiation of PDLSCs. Dentin matrix protein 1 (DMP1) and its receptor, Glucose regulated protein-78 (GRP78), are localized in the progenitor cells of the PDL. Our overall goal is to demonstrate the formation of DMP1-GRP78 complex at the plasma membrane and subsequent protein trafficking and nuclear localization to promote osteogenic differentiation. To study the internalization and routing of the complex, we mimic an *in vivo* differentiation scenario by stimulating cells with DMP1 and culturing them in the presence of osteogenic differentiation conditions. We first demonstrate the translocation of the ER chaperone protein GRP78 to the plasma membrane during the differentiation process. Total internal reflection microscopy imaging demonstrates the formation and internalization of the receptor- ligand (GRP78-DMP1) complex. Confocal microscopy results show the internalization of the GRP78-DMP1 complex specifically through the caveolin pathway and trafficked through the cell with various endocytic markers such as Rab5 and 7 GTPases to early and late endosomes respectively. DMP1 is ultimately transported to the nucleus where it functions to promote osteogenic differentiation as demonstrated by quantitative Real-Time PCR. This observation is the first report that suggests DMP1 and GRP78 can interact at the plasma membrane, then packaged in vesicles and ultimately DMP1 is routed to the nucleus where it aids in osteogenic differentiation of PDLSCs. Characterizing the osteogenic potential of PDLSCs would favor the development of therapeutic strategies for reconstruction of mineralized tissues destroyed by periodontal diseases.

## Introduction

The healthy periodontium, consisting of the gingiva, alveolar bone, periodontal ligament and cementum, functions in anchoring the tooth to the alveolar bone. In pathological conditions such as periodontal diseases, trauma or excessive force, destruction of the periodontal tissue can occur and result in loss of tooth structure in the adults (Mortazavi and Baharvand, 2016). In the human periodontal ligament (PDL), heterogeneous cell populations have been identified, among which reside a rich source of multipotent, periodontal ligament stem cells (PDLSCs). Under appropriate cues, PDLSCs are capable of differentiating into osteoblasts, cementoblasts and fibroblasts, making them a unique population of adult stem cells (Zhu and Liang, 2015). PDLSCs were used in this study to demonstrate their feasibility in promoting osteogenic differentiation as the adult stem cells from different tissues have unique epigenetic and transcription factors to regulate its function. Therefore, repair of the damaged periodontium by PDLSCs might have a potential as a cellular-based treatment. In this study, we demonstrate the osteogenic differentiation of PDLSCs by utilizing a regulatory matrix molecule DMP1 and its receptor Glucose regulated protein-78 (GRP78). Although the regeneration of bone is a complicated process, stem cells have been proven to be advantageous in developing new strategies to overcome these issues.

Dentin Matrix Protein-1 (DMP1) is a key regulatory protein in the development of an organized mineralized matrix in bone and dentin because of its ability to bind calcium and initiate mineral nucleation and growth (Butler and Ritchie, 1995; He et al., 2003). DMP1 contains a nuclear localization signal and a nuclear export signal which facilitates its transport into the nucleus and export out into the extracellular matrix (Jacob et al., 2014). Although DMP1 is a known resident of the ECM, its function in the nucleus of pre-osteoblasts play an important role in the terminal differentiation of osteoblasts. We have previously demonstrated that DMP1 in the ECM can be internalized with the aid of its receptor GRP78 localized on the plasma membrane of osteoblasts (Ravindran et al., 2008).

Glucose regulated protein-78 is an endoplasmic reticulum (ER) chaperone protein of the heat shock family that plays a dynamic role in a multitude of ER processes. When proteins are translocated to the ER during synthesis, GRP78 in the lumen binds these proteins to ensure proper folding. As a modulator of ER stress, GRP78 functions in maintaining cellular homeostasis (Lee, 2005; Li et al., 2008). In osteoblasts, GRP78 from the ER can translocate to the plasma membrane from its typical residence in the ER under stress conditions (Tsai et al., 2015) such as an increase in intracellular calcium levels. This translocation of GRP78 to the plasma membrane facilitates its binding with DMP1.

Internalization of the receptor-ligand complex from the plasma membrane can occur through several endocytic pathways namely; clathrin and caveolin mediated pathways, phagocytosis, or pinocytosis. Once the complex has been internalized, Rab GTPases can coordinate vesicular transport within the cytoplasm. The Rab family of proteins contains over 70 different proteins that can deliver the internalized complexes to their correct destination (Hutagalung and Novick, 2011). Rab5 and Rab7 are known to sort the internalized complexes into early and late endosomes respectively. Rab11, a recycling endosome, can recycle the cargo back to the plasma membrane. The internalization and transport mechanism of the GRP78-DMP1 complex from the plasma membrane to the nucleus has yet to be determined (Stenmark, 2009). Differentiation of progenitor cells to osteoblasts require transport of DMP1 from the plasma membrane to the nucleus.

In this work, we demonstrate for the first time the internalization and transport mechanisms of the DMP1-GRP78 complex using caveolin-mediated pathway and subsequent localization of DMP1 in the nucleus of PDLSCs. Understanding the intracellular transport mechanisms is key to understanding the differentiation of PDLSCs into the osteogenic lineage. Identifying the function of such key players resident in PDLSCs and their role in osteogenic differentiation of stem cells, and matrix mineralization would be necessary for development of tissue engineering strategies to regenerate the PDL or repair periodontal defects and thereby prevent tooth loss.

## Materials and Methods

### Immunofluorescence and Immunocytochemistry

One-month wild type (WT) post-natal mouse mandibles were fixed in 10% neutral buffered formalin at 4°C for 3 days prior to processing for paraffin embedding. Decalcification was performed with 10% EDTA (Ethylenediaminetetraacetic acid), pH 7.4 and confirmed with Faxitron imaging to assess the extent of demineralization. The mandibles were then paraffin embedded and 5 μm sections were processed with immunofluorescence according to published protocols (Eapen et al., 2012a). All mice related studies were performed as per UIC protocol Animal Assurance Number 16–178. The sections were probed with anti-Rab5 rabbit polyclonal or anti-Rab5 mouse monoclonal antibody (1/100; Cell Signaling Technology, Danvers, MA or 1/100; Santa-Cruz Biotechnology, Dallas, TX, United States), anti-Rab7 rabbit polyclonal or anti-Rab7 mouse monoclonal antibody (1/100; Cell Signaling Technology or 1/100; Santa-Cruz Biotechnology, Dallas, TX, United States), anti-Rab11 rabbit polyclonal or anti-Rab11 mouse monoclonal antibody (1/100; Cell Signaling Technology or 1/100 Santa-Cruz Biotechnology, Dallas, TX), anti- GRP78 mouse monoclonal (1/400; Santa-Cruz Biotechnology, Dallas, TX or 1/400; made in house), anti-DMP1 rabbit polyclonal antibody (1/100; made in house), and anti-STRO-1 mouse monoclonal antibody (Abcam, Cambridge, United Kingdom) in the various experiments. PDLSCs were seeded on glass coverslips and grown in normal growth or osteogenic differentiation media to 70–80% confluency prior to treatment with recombinant DMP1, which was made as previously described (Srinivasan et al., 1999). The hPDLSCs were stimulated with DMP1 at varying time points ranging from 5 to 60 min. Immunocytochemistry was performed as previously described (Eapen et al., 2012b). The hPDLSCs were probed with anti-Rab5, anti-Rab7, anti- Rab11, anti- Cav1 mouse monoclonal antibody (1/100; Invitrogen, Carlsbad, CA, United States), anti-GRP78, anti-DMP1, and anti-STRO-1 in the various experiments. Fluorescent goat-anti Mouse or Rabbit FITC and TRITC secondary antibodies (1/100; Sigma-Aldrich) were used, and the slides were mounted and visualized with a Zeiss 710 Meta Confocal Microscope at the UIC Core Facility. The images were analyzed through JACoP ImageJ to determine the Pearson’s Coefficient of Colocalization (PCC) with the auto-determined threshold (O’Brien et al., 2016).

### Cell Culture

Human periodontal ligament stem cells (hPDLSCs) were first isolated and characterized by Seo et al. (2004) and Mrozik et al. (2010). STRO-1, a stem cell marker, was used to confirm (Supplementary Figure S1). The hPDLSCs were cultured in α-MEM (Corning Inc., Corning, NY, United States) supplemented with 15% FBS, 1% Antibiotics, and 1% L-glutamine (Thermo Fisher Scientific, Waltham, MA, United States). For osteogenic differentiation experiments, the normal growth media was supplemented with 10 mM β-glycerophosphate (Thermo Fisher Scientific), 100 μg/mL ascorbic acid (Sigma-Aldrich, St. Louis, MO, United States), and 10 nM dexamethasone (MP Biomedicals, Santa Ana, CA, United States). Stable overexpression of GRP78 was performed by transfecting the hPDLSCs with pCDH-GRP78-GFP plasmid followed by selection with puromycin. Real-time PCR, protein expressions and fluorescent microscopy analysis confirmed the stable transfection of GRP78. Over 95% of the transfected cells expressed the GFP marker.

### TIRF Microscopy Analysis

The hPDLSCs were seeded on 35 mm collagen coated glass bottom dishes (MatTek Corporation, Ashland, MA, United States) and cultured under normal growth or osteogenic differentiation conditions. They were transiently transfected with pCDH-GRP78-GFP plasmid using DNAfectin (abm Inc., Richmond, BC, Canada), and co-transfected with CellLight Plasma Membrane marker (Thermo Fisher Scientific) according to manufacturer’s protocol. hPDLSCs were subsequently stimulated with rDMP1 at 15, 30, and 60 min and fixed with 10% neutral buffered formalin (Merkel and George, 2019). The cells were washed four times with PBS and then placed finally in PBS and then imaged. Imaging was done using a Zeiss Laser TIRF Microscope and the images were analyzed using ImageJ particle counter to count the particles within the boundaries of the plasma membrane. The cell size was averaged among the samples to obtain a normalized value of the cell size and particles.

### Inhibitor Assay

The hPDLSCs were seeded on glass coverslips in 6-well plates and grown until 60% confluent. The cells were then pretreated for 60 min with inhibitors for the clathrin-mediated pathway, Pitstop 2 (Sigma) and methyl-β-cyclodextrin (Sigma) or no inhibitor. Pitstop 2 and methyl-β-cyclodextrin (Sigma) were used at concentrations of 15 μm and 15 mM, respectively. The cells were treated with rDMP1 for 15 and 30 min then washed with PBS and fixed overnight. Immunocytochemistry was performed as previously described for DMP1 and GRP78.

### Protein Isolation and Western Blotting

Human periodontal ligament cells were grown under normal growth conditions, osteogenic differentiation conditions, rDMP1 treatment for 24 h, or rDMP1 treatment for 24 h plus osteogenic differentiation conditions. The total proteins were extracted from the hPDLSCs grown under different conditions using cell lysis reagent. For time sequence experiments, the hPDLSCs were grown for 0, 7, and 14 days in normal growth conditions and osteogenic differentiation conditions prior to total protein extraction. Twenty-five μg of total proteins were loaded on a 10% SDS-polyacrylamide gel. The proteins were transferred onto a nitrocellulose membrane after electrophoresis, blocked with 5% skim milk, and probed with anti Cav-1 rabbit polyclonal antibody (1/1000; Invitrogen), anti- GRP78 mouse monoclonal antibody (1/1000; Santa-Cruz Biotechnology), anti-Rab5 rabbit polyclonal antibody (1/1000; Cell Signaling Technology), anti-Rab7 rabbit polyclonal antibody (1/1000; Cell Signaling Technology), anti- Dynamin mouse monoclonal antibody (1/1000; R&D Systems, Minneapolis, MN, United States). Anti-Tubulin mouse monoclonal antibody (1/5000; Invitrogen) was used as a loading control. The blots were incubated in either anti-mouse or anti-rabbit secondary conjugated with HRP. Each of the blots were washed with PBS four times, and the bands were visualized using chemiluminescence detection (Thermo Fisher Scientific).

### Immunoprecipitation

Membrane proteins were extracted from hPDLSCs using the Mem-PER kit (Thermo Scientific) according to manufacturer’s protocol. Twenty-five μl of 10 mg/mL Protein A/G Magnetic Beads (Thermo Fisher Scientific, 88802) were washed four times with PBS on a magnetic bead separation rack according to the manufacturer’s protocol. The membrane fraction was combined with 10 μg of antibody, anti-DMP1 (made in house) or anti-IGG (control; Cell Signaling Technology), and the washed magnetic beads, and the solution was incubated overnight at 4°C. After overnight incubation, an additional 40 μl of beads were washed as previously described and incubated with the lysate mixture for 1 h. The beads were collected on the magnetic bead separation rack and washed four times. The beads were then boiled with 2× Laemmli Sample buffer (Bio-Rad, Hercules, CA, United States) and subsequently separated on the magnetic bead separation rack. Western Blots were performed as previously described.

### Quantitative Real Time PCR

Total RNA was extracted from harvested cells using RNeasy Plus Mini Kit (QIAGEN, Germantown, MO, United States) according to the manufacturer’s protocol. The hPDLSCs were grown in normal growth, osteogenic differentiation conditions, DMP1 treated, and DMP1 with osteogenic differentiation conditions. cDNA was synthesized with Superscript III Reverse Transcriptase and Oligo-dT primer (Thermo Fisher Scientific) for 60 min at 50°C. qPCR was carried out using FastStart Universal SYBR Green Master reagent (Roche diagnostics, Indianapolis, IN, United States) and primer pairs (Supplementary Figure S5) as needed on an ABI StepOnePlus instrument (Thermo Fisher Scientific). The gene expression levels were control. Primers were synthesized by IDT (Integrated DNA Technologies, Inc., Coralville, IA, United States).

### Statistical Analysis

For immunohistochemistry and immunocytochemistry, the experiments were performed with an *n* ≥ 3 sections. For protein analysis, the results were normalized to tubulin and the densiometric data is shown in the Supplementary Figure S4. For gene expression analysis, three separate paired *t*-tests were performed with a significance of *p* ≤ 0.05 using Excel (Microsoft, Redmond, WA, United States).

## Results

### DMP1 and GRP78 Colocalize in Various Tissues of the Mouse Mandible

**FIGURE 1:**
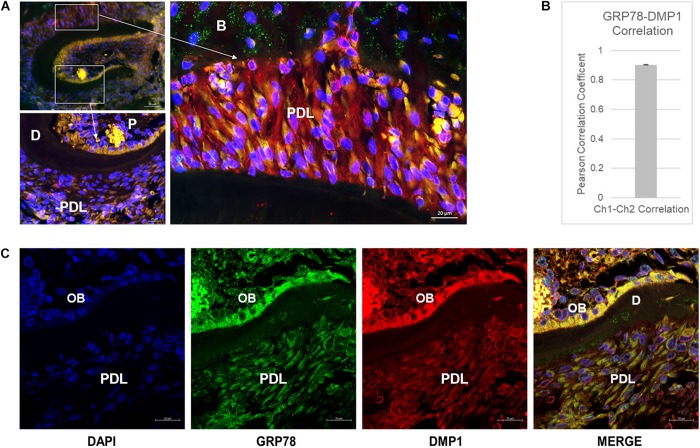
Localization of DMP1 and GRP78 in the periodontal ligament of mouse mandible. **(A)** Immunolocalization of GRP78 (FITC) and DMP1 (TRITC) and DAPI in one-month mouse mandible sections. Higher magnification of the boxed area denoted by the arrow represents the PDL and the dental pulp. P, pulp; D, dentin; PDL, periodontal ligament; B, bone. Bars represent 50 and 20 μm. **(B)** Pearson’s Correlation Coefficient between GRP78 and DMP1 in the periodontal ligament of the one-month mouse mandible sections with *n* ≥ 3 sections. **(C)** Localization of DMP1 (TRITC), GRP78 (FITC), and DAPI in one-month mouse mandibles with separated channels and the merged channel of the three colors. Co-expression of the two proteins is indicated by yellow. P, pulp; D, dentin; PDL, periodontal ligament; OB, odontoblast layer. Bars represent 10 μm.

Colocalization of GRP78 and DMP1 was observed in the PDL, odontoblasts and pulp cells in one-month old mouse mandible (Figure 1A). In the pulp, the colocalization between the two proteins is clearly observed on the cell membrane and in the cytoplasm, especially toward the odontoblast layer. In the periodontal ligament cells, colocalization was observed in the cytoplasm with GRP78 localized on the cell membrane of some cells. Pearson’s coefficient of colocalization was determined to be 0.903 indicating a strong interaction between GRP78 and DMP1 in the periodontal ligament cells (Figure 1B). Punctate staining of DMP1 is observed in the nucleus of cells in the dental pulp near the odontoblasts and around the nuclear membrane in the periodontal ligament cells (Figure 1C). In the odontoblasts, the colocalization of DMP1 and GRP78 is clearly observed Figure 1C (merged image). Thus, colocalization of DMP1 and GRP78 is observed in periodontal ligament cells, odontoblasts, and the dental pulp cells. STRO-1 staining in PDLSCs (Supplementary Figure S1) and in the developing PDL of one-month old mouse mandible (Supplementary Figure S2) was used to demonstrate the presence of stem cells.

### Translocation of GRP78 From the ER to the Plasma Membrane With DMP1 Stimulation

**FIGURE 2:**
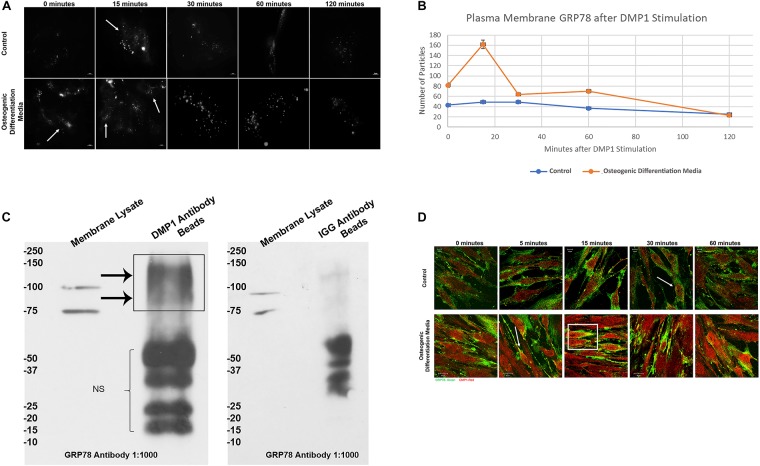
Representative TIRF Microscopy images for visualization of DMP-1 induced internalization of membrane GRP78. **(A)** PDLSCs were grown on collagen coated glass bottom dishes in either normal growth or osteogenic differentiation media and transfected with pCDH GFP-GRP78 plasmid for 48 h. The PDLSCs were stimulated with rDMP1 for 15, 30, 60, and 120 min, and the cells were visualized with TIRF microscopy to visualize the translocation of GRP78 to the plasma membrane. No stimulation with rDMP1 served as control. **(B)** Graphical representation of the number of fluorescent spots per hPDLSCs cell with rDMP1 stimulation: The spots were analyzed with Image J Particle Counter with a normalized cell area per image and subtracted background threshold. The orange line represents the number of fluorescent particles of GRP78 under osteogenic differentiation conditions with rDMP1 stimulation and the blue represents normal growth media conditions. Note the increase in GRP78 with DMP1 stimulation. **(C)** Co-immunoprecipitation of membrane bound GRP78: The membrane fraction of PDLSC overexpressing GRP78 was extracted and immunoprecipitation was performed with DMP1 antibody on Protein A/G Beads and IGG Beads as a control. The subsequent Western Blots were probed with GRP78 at a 1:1000 dilution. Arrows denote the presence of GRP78 and GRP78-GFP. **(D)** Immunofluorescence of GRP78 (TRITC-RED) and DMP1 (FITC-GREEN) in hPDLSCs after DMP1 stimulation in control and osteogenic differentiation conditions. Arrows and boxes represent areas of colocalization. Bars represent 10 μm.

Figure 2A demonstrates the translocation of GRP78 from the ER to the plasma membrane of hPDLSCs transiently transfected with pCDH-GRP78 plasmid. Upon stimulation by rDMP1, GRP78 translocates from the ER to the plasma membrane in the cells cultured under control and osteogenic differentiation conditions. Cells under osteogenic differentiation conditions showed a threefold increase of membrane GRP78 at 15 min compared to the control culture conditions (Figure 2B). The levels of GRP78 localized at the plasma membrane are highest at 15 min and then decrease with time. To demonstrate the interaction of DMP1 and GRP78 at the plasma membrane, the membrane fractions of hPDLSCs overexpressing GRP78 was isolated and immunoprecipitation was performed with DMP1 antibody or IGG antibody (control) on Protein A/G Magnetic beads. The subsequent Western Blots were probed with GRP78 to identify the interacting complex of DMP1 and GRP78. Results in Figure 2C show the presence of GRP78 in the membrane lysate (input) at 100 and 78 kDa according to the reported size of GRP78 and the GRP78 with GFP tag from the overexpressing cell line. In the sample with DMP1 beads, the band for GRP78 is seen between 75 and 100 kDa. No GRP78 bands were observed with control IGG. Confocal images in Figure 2D show that with rDMP1 stimulation at various time points, there is an increase in colocalization between DMP1 and GRP78. Both proteins are endogenously expressed, however, the stimulus of DMP1 increased the colocalization of the two proteins. Under osteogenic differentiation conditions colocalization was observed between the two proteins within 5 min when compared to normal growth conditions (Figure 2D and Supplementary Figure S3).

### DMP1-GRP78 Complex Is Internalized by the Caveolin-Mediated Endocytic Pathway

**FIGURE 3:**
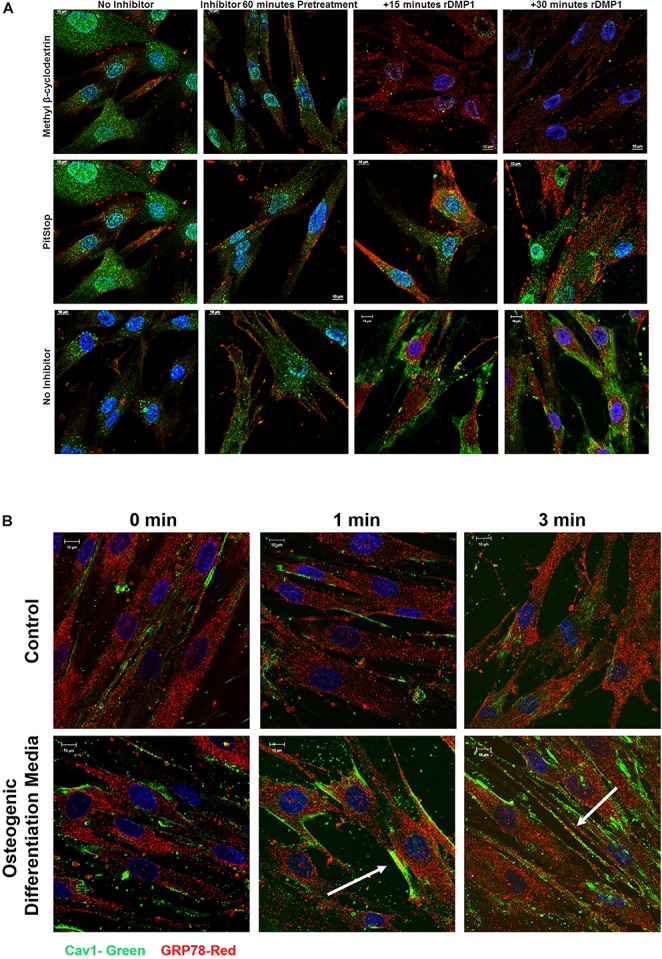
Localization of GRP78 with DMP1 stimulation in the presence of inhibitors for Caveolin and Clathrin-Mediated Endocytosis. **(A)** hPDLSCs cells were either left untreated (no inhibitor) or treated with 15 mM methyl-β-cyclodextrin, a caveolin mediated endocytosis inhibitor or 10 uM Pitstop, a clathrin mediated endocytosis inhibitor for 60 min prior to stimulation with 250 ng/ml rDMP1 at specified time points. Immunofluorescence was subsequently performed and representative confocal micrographs show the localization of DMP1 (FITC-Green) and GRP78 (TRITC-Red) along with DAPI (BLUE). Scale bar represent 10 μm. **(B)** hPDLSCs were grown in normal growth conditions and osteogenic differentiation conditions prior to treatment with rDMP1 for 10 min. Immunofluorescence was performed and representative confocal images show localization of GRP78 (TRITC-RED) and Cav-1 (FITC-GREEN). DAPI is shown in blue. Scale bar: 10 μm.

In order to ascertain the endocytic pathway by which the complex is internalized, inhibitors of both caveolin and clathrin pathways were tested. Therefore, hPDLSCs were pretreated with methyl-β-cyclodextrin (caveolin-mediated endocytosis) and Pitstop (clathrin-mediated endocytosis) and stimulated with rDMP1. Results in Figure 3A demonstrate that when cells were inhibited with methyl-β-cyclodextrin, the levels of internalized DMP1 (FITC-green) decreased at both 15 and 30 min upon rDMP1 stimulation as determined with a Pearson’s Correlation Coefficient (0.9997 pre-treatment, 0.8499 after 15, and 0.7656 after 30 min). Cells inhibited with Pitstop 2 showed increased levels of DMP1 at both 15 and 30 min of rDMP1 stimulation as determined with a Pearson’s Correlation Coefficient (0.786 pre-treatment, 0.7953 after 15 min, and 0.8346 after 30 min). Control cells with no inhibitor and with DMP1 stimulation showed increased levels of DMP1 and GRP78. In Figure 3B, hPDLSCs were treated with rDMP1 for 1 and 3 min in both normal growth and osteogenic differentiation conditions. The hPDLSCs were then immunostained with GRP78 and Caveolin-1 antibody to determine their temporal and spatial localization after treatment. After 1 min there is an increase in the colocalization of GRP78 (Red) and Cav-1 (FITC) under osteogenic differentiation conditions with colocalization observed at the plasma membrane continuing into 3 min of rDMP1 stimulation. This suggests that during cellular differentiation of PDLSCs, DMP1-GRP78 complex is internalized by the caveolin-mediated endocytic pathway.

### Endocytic Trafficking of DMP1-GRP78 Complex Is Facilitated by the Ras Superfamily of GTPases

**FIGURE 4:**
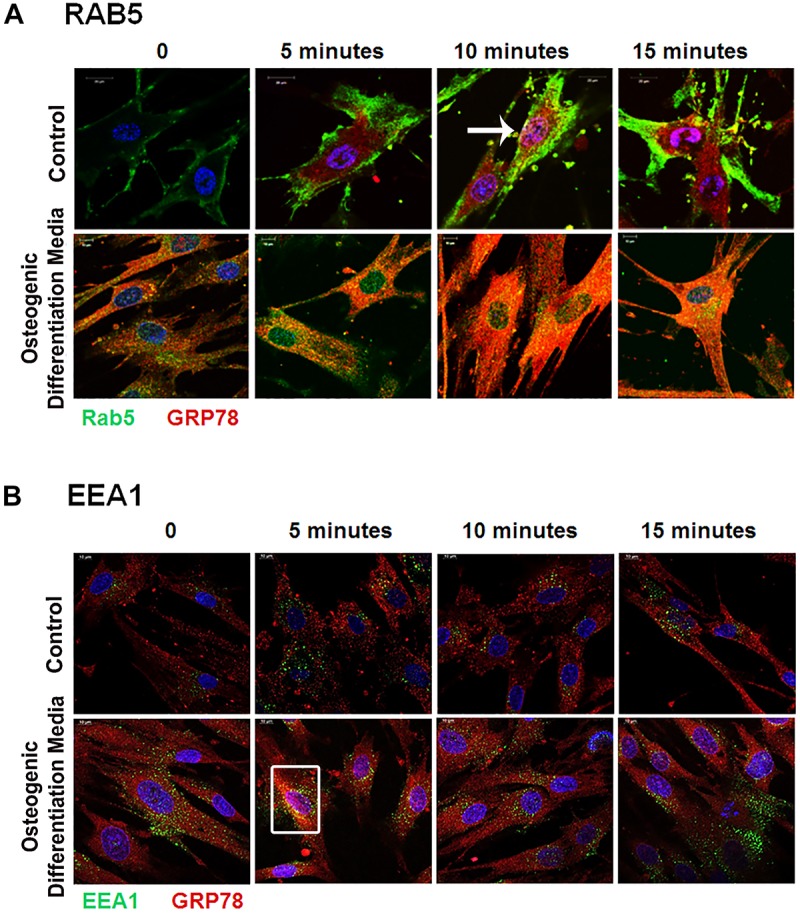
Vesicular trafficking of GRP78 with DMP1 stimulation facilitated by early endocytic mediators: hPDLSCs were subjected to osteogenic differentiation or control media for 2 days prior to treatment with rDMP1. Both groups were grown to 70–80% confluency and then serum starved for 4 h prior to treatment with rDMP1. **(A)** Representative confocal images show localization of Rab5 (FITC-GREEN), GRP78 (TRITC-RED), and DAPI (BLUE). **(B)** Representative confocal images showing localization of EEA1 (FITC-Green) and GRP78 (TRITC-Red) Confocal imaging was performed at the UIC Microscopy Core with a Zeiss Meta 710 Confocal Microscope. Arrows and boxes denote representative areas of colocalization between GRP78 and Rab5 and EEA1. Scale Bar = 10 μm.

**FIGURE 5:**
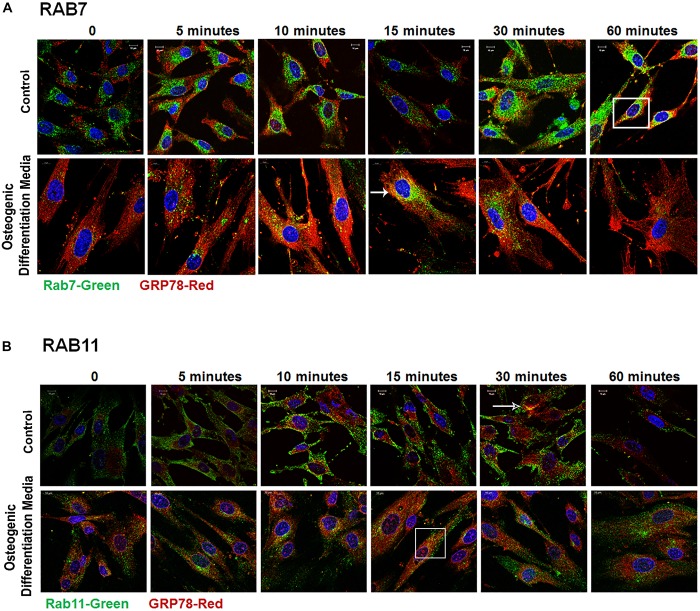
Late Endocytic Mediators of GRP78 and DMP1 Internalization. hPDLSCs were subjected to osteogenic differentiation or control media for 2 days prior to treatment with rDMP1. Both groups were grown to 70–80% confluency and serum starved for 4 h prior to treatment with rDMP1. **(A)** Representative confocal images showing localization of Rab7 (FITC-Green) and GRP78 (TRITC Red). **(B)** Localization of Rab11 (FITC-Green) and GRP78 (TRITC Red). Images were acquired with a Zeiss Meta 710 Confocal Microscope. Arrows denote areas of colocalization between GRP78 and Rab7 or 11. Scale bar = 10 μm.

**FIGURE 6:**
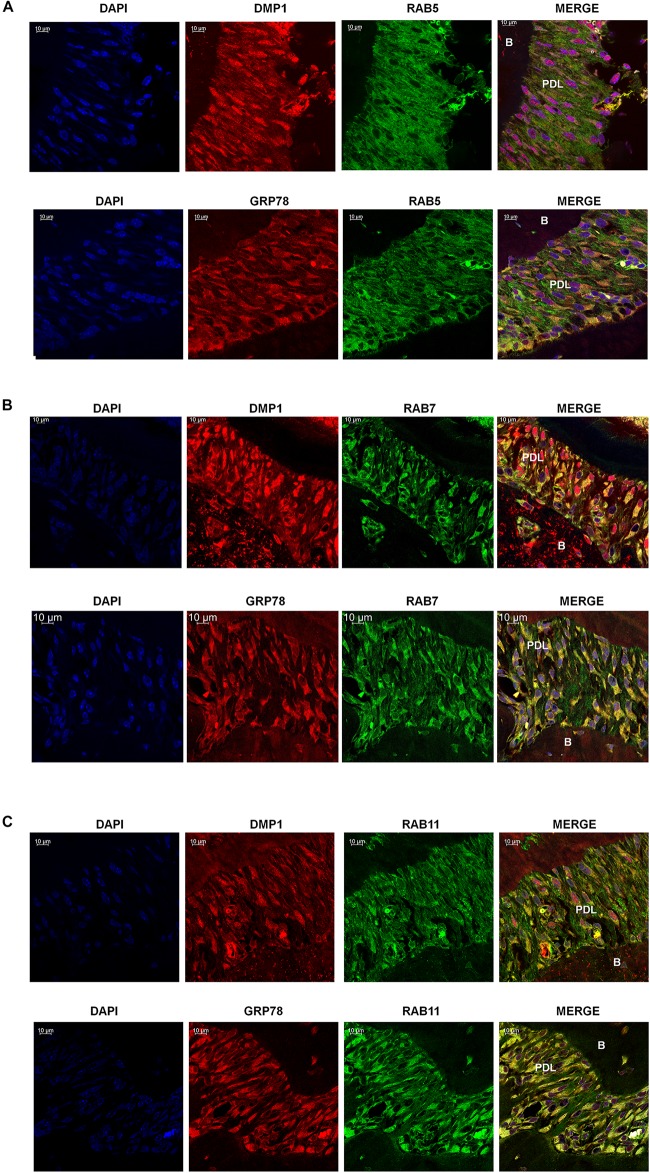
Localization of GRP78, DMP1 and the endocytic mediators in the periodontal ligament of mouse mandible. **(A)** Localization of Rab5, (FITC-Green) with DMP1 or GRP78 (TRITC-Red) in the periodontal ligament of one-month post-natal wild-type mice. **(B)** Localization of Rab7 (FITC Green) with DMP1 or GRP78 (TRITC-Red) in the periodontal ligament of one-month post-natal wild-type mice. **(C)** Localization of Rab11, (FITC-Green) with DMP1 or GRP78 (TRITC-Red) in the periodontal ligament of one-month post-natal wild-type mice. In panels **(A–C)** co-expression of two-proteins is indicated by yellow and DAPI is blue. Images were acquired with a Zeiss Meta 710 Confocal Microscope. In which panel the individual channels are shown along with the merged image. PDL, periodontal ligament, B, Bone. Scale bar = 10 μm.

Rab GTPases have been identified as the molecular machinery that coordinate vesicular trafficking through the cytoplasm and direct the endosomal cargo to the correct subcellular localization. In Figure 4, immunocytochemistry was performed on hPDLSCs stimulated with rDMP1 and immunostained with anti-GRP78 antibody and Rab5 an early endosomal marker (Figure 4A). In normal growth conditions, there is little to no colocalization between GRP78 and Rab5 until 10 and 15 min of DMP1 stimulation where the colocalization of the two proteins is found near the plasma membrane. In osteogenic conditions, colocalization is seen between GRP78 and Rab5 at all-time points with a diffuse, rather than a punctate staining pattern. Colocalization is predominantly observed at the plasma membrane and in the cytoplasm. Immunolocalization of EEA1, a Rab5 effector protein, with GRP78 show that at 5 min of DMP1 stimulation under osteogenic conditions, colocalization is observed (Figure 4B). Rab 7, a late endosomal marker, had a similar localization pattern as Rab5 as shown in Figure 5A. Colocalization between GRP78 and Rab7 occurs in 30 and 60 min after rDMP1 stimulation in normal growth conditions. In osteogenic differentiation conditions, the colocalization between GRP78 and Rab7 begins at 10 min and continues to increase in the cytoplasm and the nuclear membrane region of the PDLSCs (Figure 5A). Rab 11, a recycling GTPase marker, is seen to colocalize with GRP78 in both osteogenic differentiation and control conditions at 10–30 min (Figure 5B). This association suggests GRP78 has a potential to be recycled back to the plasma membrane by Rab11. *In vivo* studies of one-month post-natal WT mouse mandibles show the colocalization between the Rab proteins (FITC) and DMP1 (Red) in the cells of the periodontal ligament in Figure 6. Rab5 (Figure 6A) and Rab11 localization are diffuse throughout the periodontal ligament cells, while Rab7 (Figure 6B) mainly localizes to the cytoplasmic region and membrane of the cells in the PDL. Colocalization (merged Image) is seen between all the Rab proteins with DMP1, however, with Rab7 and Rab11 (Figure 6C) showed higher colocalization levels when compared to Rab5. The colocalization of Rab and GRP78 in the mouse mandibles shows similar patterns of localization as DMP1 and Rab proteins, suggesting that both DMP1 and GRP78 are in endocytic vesicles transported by the Rab proteins.

### Osteogenic Differentiation Conditions Increases Gene and Protein Expression of Endocytic Markers

**FIGURE 7:**
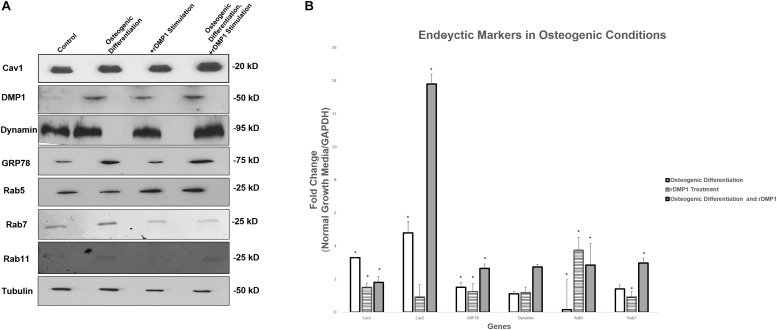
Protein and Gene Expression Analysis of GRP78, DMP1 and its Endocytic Regulators. **(A)** Total proteins were isolated from PDLSCs (control); PDLSCs grown under osteogenic differentiation conditions; PDLSCs stimulated with DMP1 for 24 h; PDLSCs stimulated with DMP1 for 24 h and grown under osteogenic differentiation conditions for 2 days. Western blots analysis was performed with antibodies against Cav1, DMP1, Dynamin, GRP78, Rab5, and Rab7. Equal loading of the proteins was confirmed by loading with Tubulin. **(B)** Total RNA was isolated from hPDLSCs in normal growth conditions and two-day osteogenic differentiation conditions and subjected to quantitative real-time PCR. The results were normalized to one. Fold change was determined with respect to the control which was normalized as 1. Values are the mean ± standard deviation of triplicate samples. A statistically significant difference is denoted with ^∗^*P* < 0.05 vs. control. Comparisons were performed using the Student’s *t*-Test.

Results in Figure 7A suggests that Cav1, the protein involved in caveolin-mediated endocytosis, is up-regulated modestly in osteogenic differentiation conditions with DMP1 stimulation compared to the control. DMP1 is found in low levels in the control conditions, however, under osteogenic differentiation conditions a sharp increase in DMP1 levels was observed. GRP78 is greatly increased by osteogenic differentiation conditions, and both Rab 7 and Rab 11 are found predominantly in the osteogenic differentiation conditions. Interestingly, Rab5 is the only protein to become up-regulated under osteogenic conditions and with DMP1 stimulation. Gene expression for endocytic regulators showed an upregulation of Rab5, Rab7, Cav1, Cav2, and dynamin similar to the protein expression. Rab5 has a 3.5-fold increase in normal growth conditions with DMP1 stimulation, suggesting that DMP1 could be an early stimulus for the downstream endocytic events (Figure 7B). The genes with a star were significantly increased with a *p*-value less than 0.05.

### Osteogenic Differentiation Conditions Promote Differentiation of PDLSCs Into Osteoblast-Like Cells

**FIGURE 8:**
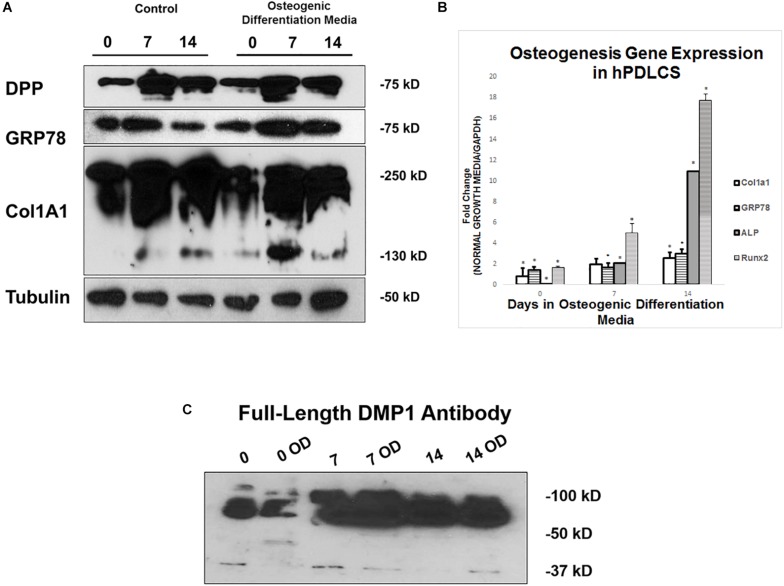
Differentiation of hPDLSCs cultured in Control and Osteogenic Conditions. **(A)** hPDLSCs were grown for 0, 7, and 14 days in control or osteogenic differentiation conditions prior to total protein extraction. Western Blots were developed with antibodies against DPP, GRP78, Col1a1, and Tubulin as a loading control. **(B)** hPDLSCs were grown for 0, 7, and 14 days in control or osteogenic differentiation conditions prior to total RNA extraction. qPCR was performed with osteogenic gene markers. The results were normalized to hGAPDH. Fold change was determined with respect to the control which was normalized as 1. **(C)** hPDLSCs were grown for 0, 7, and 14 days in control or osteogenic differentiation conditions (OD) prior to total protein extraction. Western Blots were developed with antibodies against full length DMP1. Values are the mean ± standard deviation of triplicate samples. A statistically significant difference is denoted with ^∗^*P* < 0.05 vs. control. Comparisons were performed using the Student’s *t*-Test.

hPDLSCs were grown in control and osteogenic differentiation conditions for 0, 7, and 14 days before they were harvested for protein and gene expression analysis. Protein expression analysis demonstrate that DPP and GRP78 both increase in osteogenic differentiation conditions with 7 days of differentiation being the largest increase (Figure 8A). Processing of procollagen to mature collagen is more pronounced under differentiation conditions when compared with the control. The processed collagen was seen as early as 7 days under differentiation conditions. Osteogenic gene expression analysis in Figure 8B show that under differentiation conditions, there is an upregulation of osteogenic markers ALP, Runx2, and Col1a1 at both 7 and 14 days. In Figure 8C, DMP1 expression levels are higher in osteogenic differentiation conditions and the protein is processed differently in osteogenic differentiation with bands seen at 50 kDa vs. 37 kDa.

## Discussion

Periodontitis is a highly widespread oral disease that if left untreated results in loss of PDL, cementum and bone ultimately leading to bone structure and tooth loss. It is also associated with several systemic diseases (Kinane and Marshall, 2001). Seo et al. (2004) have identified adult post-natal mesenchymal stem cells called the periodontal ligament stem cells in the PDL that have regenerative capacity. Specifically, these stem cells are multipotent and can differentiate into osteoblasts, cementoblasts, adipocytes, chondrocytes or fibroblasts in the presence of appropriate signaling cues, thus making it an attractive cell type for repairing periodontal defects. Published reports have shown that DMP1 and GRP78 are necessary for differentiation of preosteoblast cells into functional osteoblasts (Ravindran et al., 2008). Therefore, in this study, our focus is to understand the spatial localization of DMP1 and its receptor GRP78 under growth and osteogenic differentiation conditions and to identify the intracellular transport mechanisms during differentiation of PDLSCs into osteogenic lineage. Understanding such mechanisms would help in designing therapeutic strategies for periodontal bone loss.

Dentin matrix protein 1 is a key regulatory protein required for the proper calcification of the extracellular matrix in bone and teeth. Mutations in this known extracellular matrix protein are prominent in diseases, such as osteomalacia and rickets (Feng et al., 2006). DMP1 also functions intracellularly to aid in the differentiation of pre-osteoblasts and pre-odontoblasts by acting as a transcriptional regulator in the nucleus, suggesting that DMP1 has dual functions in biomineralization (Qin et al., 2007; Siyam et al., 2012; Jacob et al., 2014).

Glucose regulated protein-78 is often known for its functions in the ER and considered as the master regulator of ER homeostasis. Studies have shown that GRP78 can also function outside of ER (Pfaffenbach and Lee, 2011). Cell stress can activate the Unfolded Protein Response (UPR), leading to GRP78’s activation and allow downstream signal transduction events for cell survival or apoptosis (Lee, 2005; Deng et al., 2013). This response can allow GRP78 to reside in other parts of the cell, such as the plasma membrane or in the ECM. Plasma membrane GRP78 is found in stressed cancer cells and published reports demonstrate the use of plasma membrane GRP78 as a biomarker for cancer therapy (Tsai et al., 2015; Tsai and Lee, 2018).

In this study, we have identified the phenotypic effects of DMP1 stimulation on PDLSCs and their effect on osteogenic differentiation. Through total internal reflection microscopy, a technique that visualizes events at the plasma membrane, we demonstrated that rDMP1 stimulation and osteogenic differentiation conditions increases the translocation of GRP78 from the ER to the plasma membrane of PDLSCs. Additionally, with prolonged stimulation, presence of plasma membrane GRP78 peaks at 15 min and decreases after, suggesting that the major endocytic events are early events that are initiated upon rDMP1 stimulation in both control and osteogenic differentiation conditions. Translocation of GRP78 to the plasma membrane could be an active process in biomineralization as cells involved in the synthesis of a calcified matrix handle an influx and efflux of calcium that could act as a stress trigger for GRP78 translocation to the plasma membrane (Ravindran et al., 2008). Colocalization of DMP1 and GRP78 was also observed intracellularly in the odontoblasts and the cells of the PDL of one-month old mice mandible suggesting the close proximity of these two proteins *in vivo*. Thus, the intimate relationship between GRP78 and DMP1 might be necessary for vesicular transport of the complex and nuclear localization of DMP1. Nuclear localization of DMP1 could be an early event that might be necessary for the differentiation of PDLSC into functional osteoblast-like cells and formation of a mineralized matrix.

Understanding the endocytosis pathway and transport of the DMP1-GRP78 complex is essential to understand the potential of PDLSCs to promote osteogenesis. Using the inhibitor of both the clathrin mediated pathway, Pitstop, and the caveolin-mediated pathway, methyl-β-cyclodextrin, we show that upon rDMP1 stimulation, the levels of DMP1 decrease in the methyl-β-cyclodextrin treated samples. With treatment of the clathrin-inhibitor, the levels of rDMP1 increased as expected, suggesting that the endocytosis of the DMP1-GRP78 occurs via the caveolin-mediated endocytic pathway (Dutta et al., 2012). Caveolin proteins, Cav-1-3, are known to be involved in stem cell proliferation, differentiation and osteogenesis. A knockout mouse of Cav1 has shown a decrease in osteoclastogenesis, showing a role in bone metabolism (Razani et al., 2001). Caveolin proteins, specifically Cav1-2, are found on the membrane of osteoblasts (Lofthouse et al., 2000) that supports this evidence that DMP1 and GRP78 enter via the caveolin endocytic pathway to regulate the expression of matrix proteins.

It is necessary for cargo entering the cell through the caveolin pathway to become sorted to its proper subcellular localization. The Rab family of proteins are small GTPases that act as molecular machinery that regulates vesicular trafficking and deliver cargo to specific subcellular compartments. Studies on osteoblast differentiation have shown that upon ascorbic acid treatment, there is an up-regulation of Rab GTPases to facilitate the process of collagen trafficking in the cell (Hagiwara et al., 2009). Specifically, Rab5, an early endosomal marker, was shown to interact directly with caveolin-1 protein to mediate entry of collagen into cells. In this study, we demonstrate that the DMP1-GRP78 containing vesicles is internalized through the caveolin pathway, and Rab5 directs vesicle transport to early endosomes as demonstrated by EEA1 an early endosomal marker and a Rab5 effector, at early time points after stimulation in both control and osteogenic differentiation conditions. These early endocytic events are more prominent in the osteogenic differentiation conditions, suggesting that Rab5/EEA1 vesicle trafficking is an intracellular process during osteogenic differentiation of PDLSCs (Christoforidis et al., 1999). Interestingly, Rab5 protein levels were increased upon DMP1 stimulation, suggesting that this stimulus aids in the early events of DMP1 internalization. Endosome maturation involves Rab5 and Rab7 to help coordinate the transition from an early endosome to late endosome (Hagiwara et al., 2009). Rab7, a late endosomal marker, has been shown to be involved in the maintenance of bone homeostasis. Studies have shown that downregulation of Rab7 in osteoclasts results in impaired bone resorption (Zhao et al., 2001). Understanding the localization of Rab7 is important for osteoblast differentiation and bone formation. Localization of Rab7 with GRP78 after DMP1 stimulation, suggests that DMP1-GRP78 complex is shuffled through the cytoplasm from the early to late endosomes and that Rab7 plays a role in PDLSCs. Rab11, a recycling endosome, was found to colocalize with GRP78 demonstrating that DMP1 and GRP78 can be recycled to the plasma membrane to its destination in the extracellular matrix. Like Rab7, downregulation of Rab11 results in impaired bone resorption, suggesting the importance of Rab proteins in proper bone formation (Zhao et al., 2001).

Formation of the mineralized matrix involves both intracellular trafficking of DMP1 as well as its function in the extracellular matrix. Here in this study, we demonstrate that osteogenic conditions along with DMP1 stimulation can stimulate the differentiation of PDLSCs to osteogenic lineages with the increased expression of the “master” transcription factor Runx2, collagen 1 and ALP (Westhrin et al., 2015). These osteogenic genes can promote osteoblast differentiation with secretion of a proper organic matrix predominantly containing type I collagen which is a necessary template for the formation of the mineralized matrix (Hao et al., 2009). Dentin phosphoprotein (DPP), another member of the SIBLING family, is also an extracellular matrix protein that binds calcium and promotes hydroxyapatite formation on collagen fibrils (Eapen et al., 2012a). We and others have demonstrated the role of DMP1 in the formation of mineralized tissues (Feng et al., 2006; George et al., 2018). In the PDLSCs, we have observed DMP1 expression in both growth and osteogenic differentiation conditions, however, the processing pattern is pronounced under differentiation conditions. It has been demonstrated that the proteolytic processing of DMP1 is an activation step that releases functional fragments from the inactive full-length precursor during biomineralization. Under physiological conditions DMP1 is processed into a 37 kDa N-terminal fragment and the 57 kDa C-terminal fragment. We have previously demonstrated that the C-terminal fragment is transported to the extracellular matrix of bone and dentin where it participates in matrix mineralization. Using mouse models Ye et al., showed that deletion of DMP1 lead to an increased susceptibility to periodontal diseases in mice, suggesting that DMP1 is essential for the formation and maintenance of a healthy periodontium (Sun et al., 2011).

**FIGURE 9:**
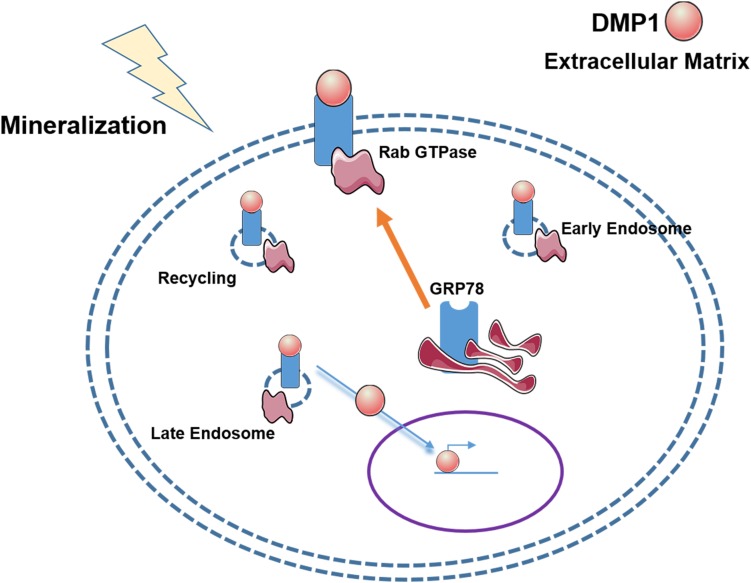
Hypothetical Model of the vesicular trafficking of DMP1-GRP78 during endocytosis. The model shows that under stress conditions or a mineralization stimulus, GRP78 translocates from the ER to the plasma membrane to act a receptor for DMP1. The DMP1-GRP78 complex translocates intracellularly and moves from the early endosome to the late endosome with the help of Rabs 5 and 7, respectively, and recycling of GRP78 by Rab11. Nuclear translocation of DMP1 from the cytoplasm could be facilitated by importin alpha. In the nucleus, DMP1 can help with the transcription of genes responsible for osteoblast/odontoblast differentiation.

Osteoblast differentiation from mesenchymal stem cells such as PDLSCs requires internalization of DMP1 along with its receptor GRP78 through well-defined internalization and transport mechanisms (Figure 9). Endocytosis of the DMP1-GRP78 complex using caveolin and Rab GTPases demonstrate the well-defined events orchestrated by stem cells during osteogenic differentiation process. Nuclear translocation of DMP1 is a prerequisite for osteogenic gene expression. The proteins could then be recycled to the plasma membrane through Rab11 and exported to the extracellular matrix to aid in mineralized matrix formation. Understanding the mechanisms by which DMP1 a signaling molecule aids in the transformation of PDLSCs into osteoblasts would greatly benefit in the regeneration of periodontal tissues such as alveolar bone or cementum.

## Data Availability

The datasets generated for this study will not be made publicly available. Fewer than 20 genes were analyzed.

## Ethics Statement

The animal study was reviewed and approved by UIC Animal Care Committee (Assurance number 16-178).

## Author Contributions

AM performed the experiments, assembled the data, and wrote the manuscript. YC helped with experimental design and data analysis. AG analyzed the data and edited the manuscript.

## Conflict of Interest Statement

The authors declare that the research was conducted in the absence of any commercial or financial relationships that could be construed as a potential conflict of interest.
