# Supplementary material for: Endocytic Trafficking of DMP1 and GRP78 Complex Facilitates Osteogenic Differentiation of Human Periodontal Ligament Stem Cells
Source: Front Physiol. 2019 Sep 12;10:1175. doi: 10.3389/fphys.2019.01175 (PMC6751249; doi:10.3389/fphys.2019.01175)
Supplement: Supplementary file 1 [file Data_Sheet_1.docx]

**Supplemental Figures:**

**Figure 1: STRO-1 (FITC) and DAPI Staining of hPDLSCs**


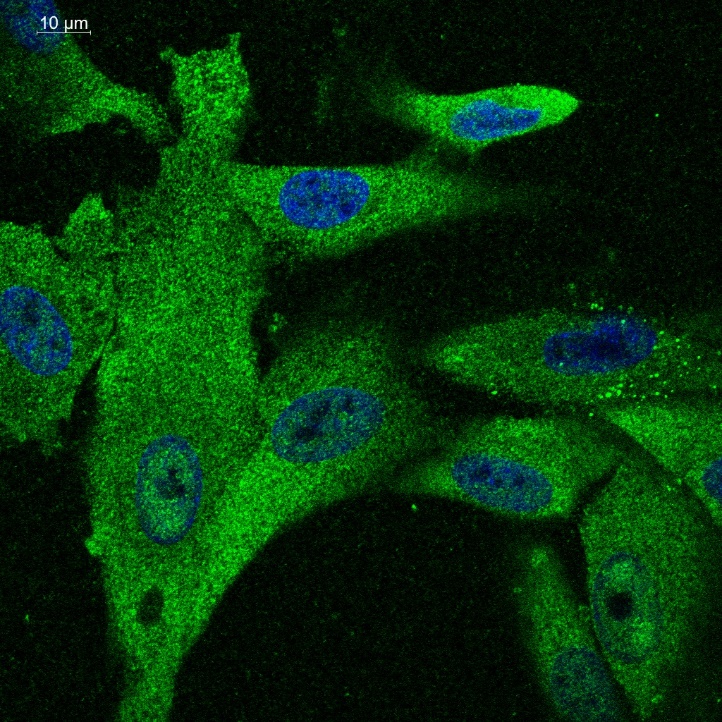


Figure 1: STRO-1 (FITC) and DAPI staining of hPDLSCs. Bar= 10 μM

**
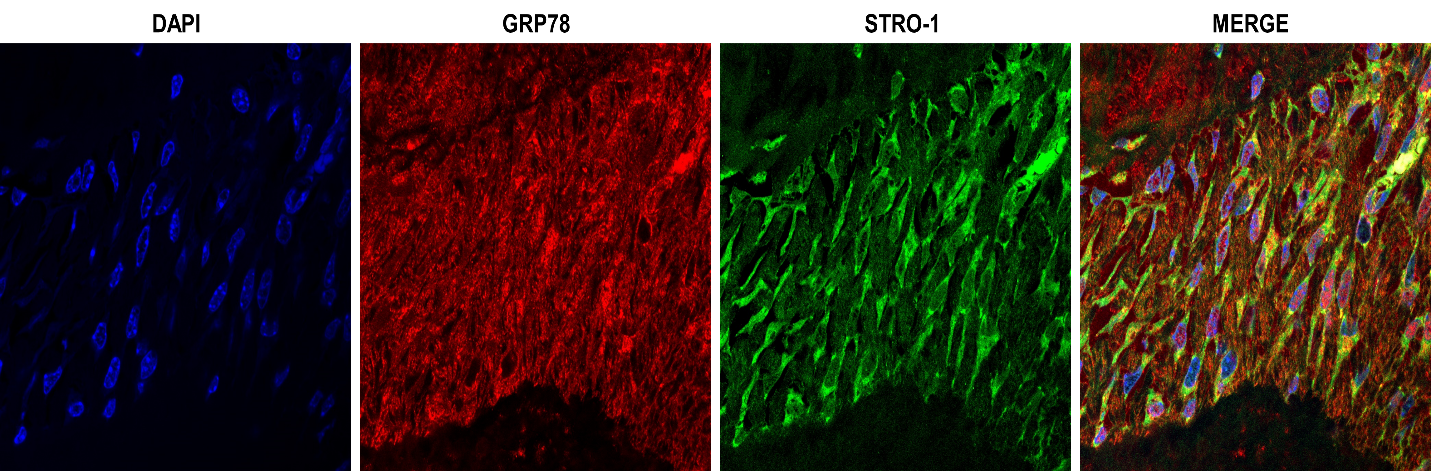
Figure 2: STRO-1 (FITC) and GRP78 in the PDL *in vivo***

Figure 2: STRO-1 and GRP78 in the PDL of one month post-natal WT mice. Imaged here is the PDL.

**Figure 3: Separated and Merged Channels for Figure 2D**

**Control**

**
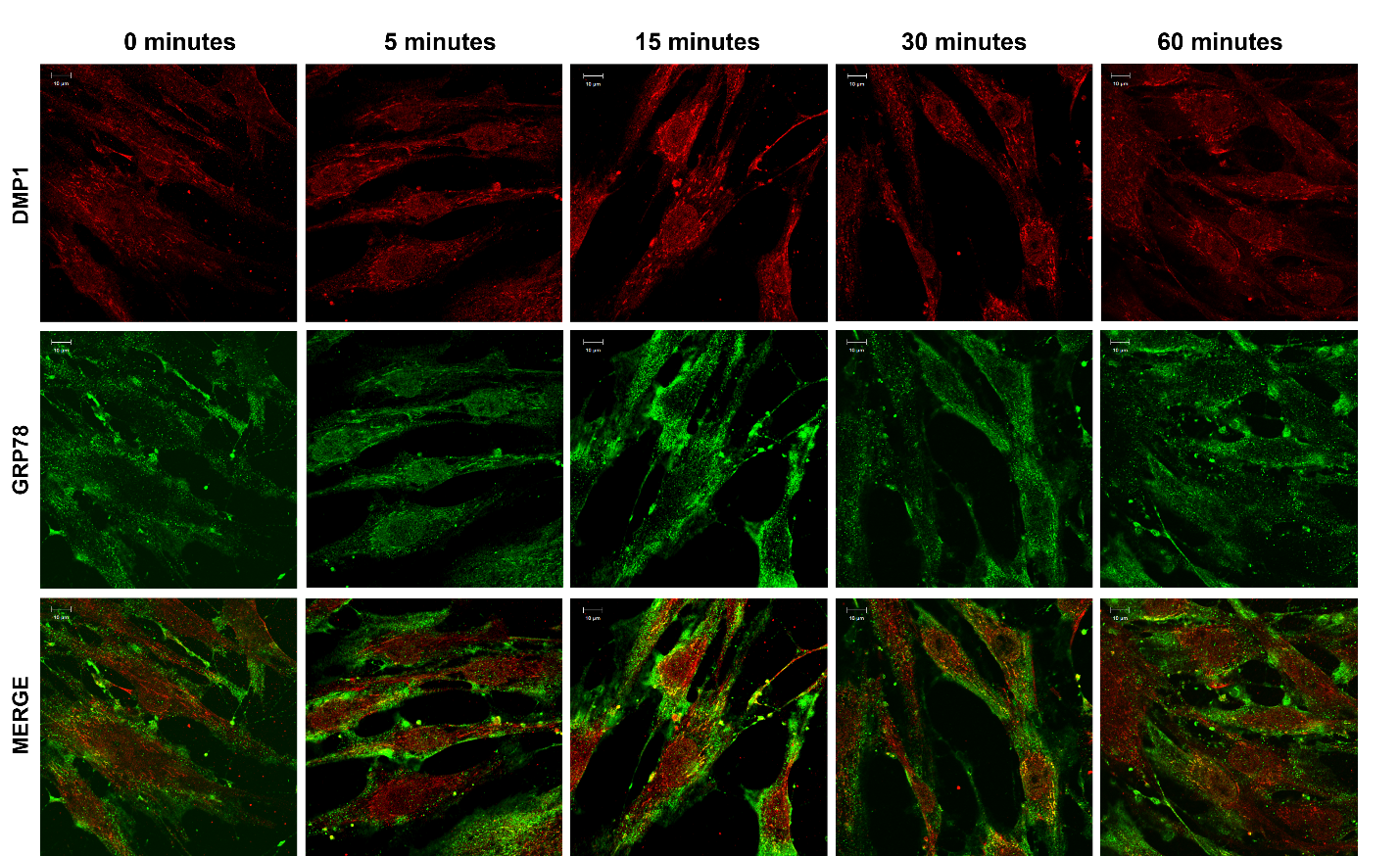
**

**
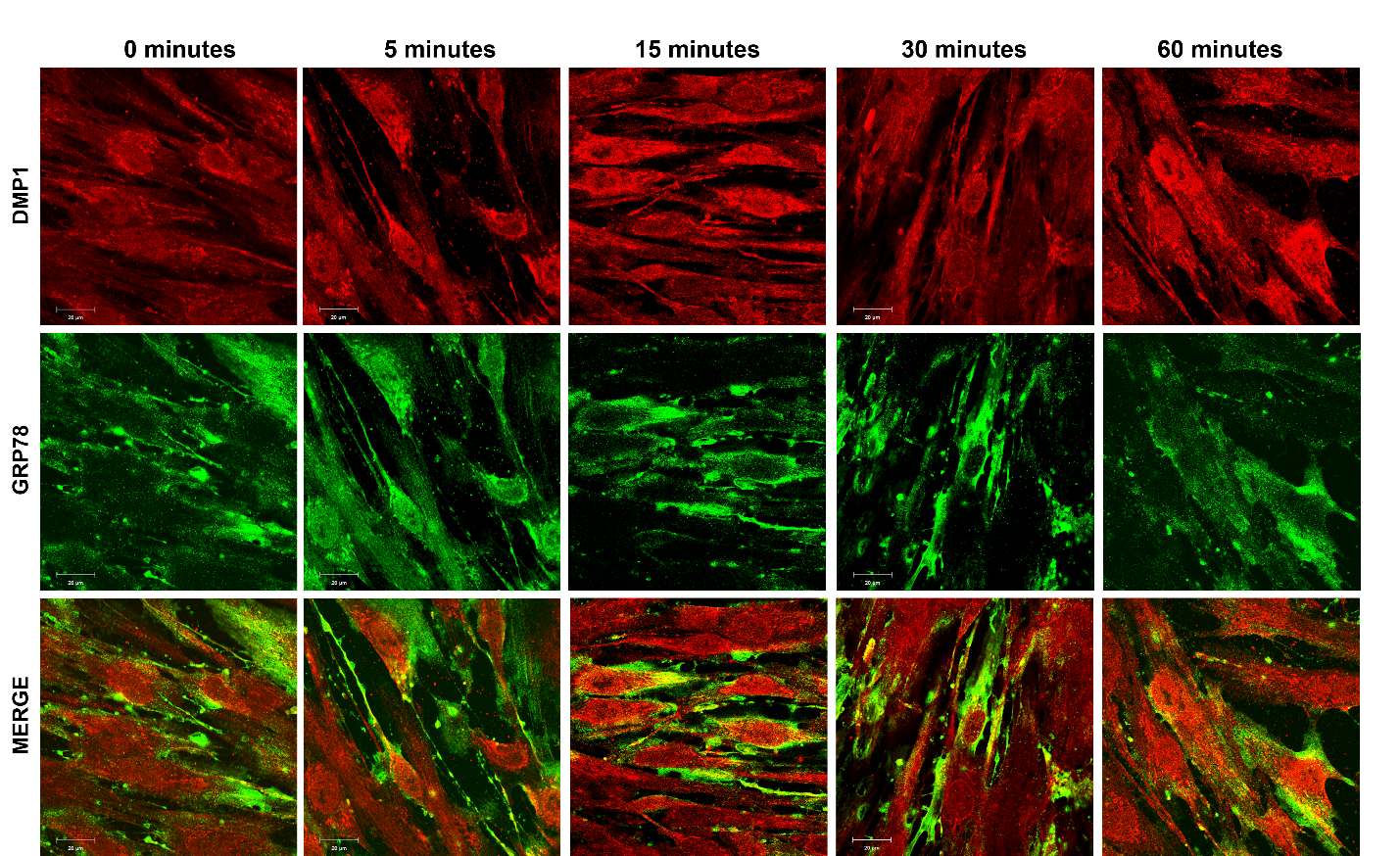
**

**Osteogenic Differentiation Media**

**Figure 4: Density ratio of protein expression normalized to Tubulin for Figures 7A and 8A**

**Figure 5:**

**Primer Table**

| **Gene** | **Forward (5’-3’)** | **Reverse (5’-3’)** |
| --- | --- | --- |
| **Rab5** | **ACGGGCCAAATACGGGAAAT** | **TCAAACTTTACCCCAATGGTACTC** |
| **GAPDH** | **GGATTTGGTCGTATTGGG** | **GGAAGATGGTGATGGGATT** |
| **Rab7** | **CAGACAAGTGGCCACAAAGC** | **AAGTGCATTCCGTGCAATCG** |
| **Cav1** | **CGACCCTAAACACCTCCACGA** | **TAAATGCCCCAGATGAGTGC** |
| **Cav2** | **ATGCCCTCTTTGAAATCAGC** | **CTCGTACACAATGGAGCAAT** |
| **Dynamin** | **AGGTCCCTTTTCATCCCAACT** | **GCTGGCATAGTCTCTAGTCCT** |
| **GRP78** | **CATCCAGCCGTCCTATGTGC** | **CGTCAAAGACCGTGTTCTCG** |
| **ALP** | **GTGCCAGAGAAAGAGAGAGA** | **TTTCAGGGCATTTTTCAAGGT** |
| **RUNX2** | **CCTGAACTCTGCACCAAGTC** | **GAGGTGGCAGTGTCATCATC** |
| **COL1A1** | **GAGGGCCAAGACGAAGACATC** | **CAGATCACGTCATCGCACAAC** |
